# Scanning Ultrasound (SUS) Causes No Changes to Neuronal Excitability and Prevents Age-Related Reductions in Hippocampal CA1 Dendritic Structure in Wild-Type Mice

**DOI:** 10.1371/journal.pone.0164278

**Published:** 2016-10-11

**Authors:** Robert John Hatch, Gerhard Leinenga, Jürgen Götz

**Affiliations:** Clem Jones Centre for Ageing Dementia Research, Queensland Brain Institute, The University of Queensland, St Lucia Campus, Brisbane, QLD 4072, Australia; Bilkent University, TURKEY

## Abstract

Scanning ultrasound (SUS) is a noninvasive approach that has recently been shown to ameliorate histopathological changes and restore memory functions in an Alzheimer's disease mouse model. Although no overt neuronal damage was reported, the short- and long-term effects of SUS on neuronal excitability and dendritic tree morphology had not been investigated. To address this, we performed patch-clamp recordings from hippocampal CA1 pyramidal neurons in wild-type mice 2 and 24 hours after a single SUS treatment, and one week and 3 months after six weekly SUS treatments, including sham treatments as controls. In both treatment regimes, no changes in CA1 neuronal excitability were observed in SUS-treated neurons when compared to sham-treated neurons at any time-point. For the multiple treatment groups, we also determined the dendritic morphology and spine densities of the neurons from which we had recorded. The apical trees of sham-treated neurons were reduced at the 3 month time-point when compared to one week; however, surprisingly, no longitudinal change was detected in the apical dendritic trees of SUS-treated neurons. In contrast, the length and complexity of the basal dendritic trees were not affected by SUS treatment at either time-point. The apical dendritic spine densities were reduced, independent of the treatment group, at 3 months compared to one week. Collectively, these data suggest that ultrasound can be employed to prevent an age-associated loss of dendritic structure without impairing neuronal excitability.

## Introduction

Recently, our group has reported that repeated scanning ultrasound (SUS) treatments reduced the amyloid plaque pathology in the APP23 transgenic mouse model of Alzheimer's disease (AD) and improved hippocampal-dependent spatial memory performance by activating brain-resident microglia [1]. In this approach, ultrasound was combined with microbubbles to disrupt the blood-brain barrier (BBB) which is achieved by mechanical interactions between the microbubbles and the blood vessel wall as pulsed focused ultrasound is applied, resulting in cycles of compression and rarefaction of the microbubbles [2, 3]. This leads to a transient disruption of tight junctions and the uptake of blood-borne factors by the brain [4], which are likely to have a role in the activation of microglia that were found to take up amyloid into their lysosomes [1, 5].

**Fig 1:**
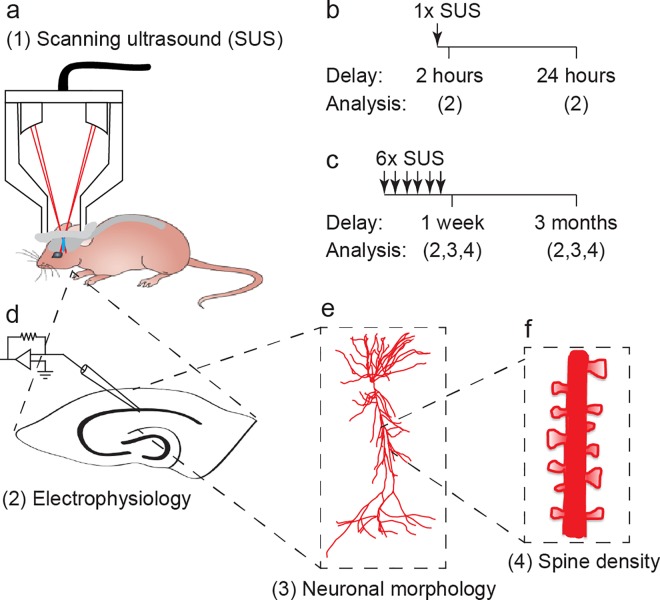
Overview of experimental study design. (a) Schematic of scanning ultrasound (SUS) setup. (b-f) Treatment and experimental scheme. A cohort of wild-type mice was treated with (b) a single SUS treatment, and (d) electrophysiological recordings were performed 2 hours or 24 hours later to investigate the acute affects of SUS on neuronal excitability. A second cohort of mice was treated with (c) six SUS treatments once per week for six weeks and allowed to age for one week or three months before (d) electrophysiology, (e) neuronal morphology and (f) dendritic spine density were investigated to determine how SUS treatment affects neuronal excitability and synaptic connectivity.

If one intends to explore the ultrasound technology for therapeutic applications, safety is an obvious concern [2, 6]. Fortunately, there are several studies that imply that a bio-effect can be achieved in the absence of overt damage. One of the reasons for this is that ultrasound is highly tunable, and when its parameters are carefully chosen, BBB opening can be achieved without causing overt histological damage as shown in both transgenic AD mouse models and wild-type mice [1, 5, 7], but also larger animals such as macaques [8, 9]. Importantly, it has been reported that treatment with ultrasound for up to 20 months in non-human primates does not alter neurological functions, including visual, cognitive, motivational and motor functions [10]. However, the short- and long-term effects of SUS treatment on individual neuronal action potential (AP) firing and dendritic morphology have not been investigated. To address this issue, we evaluated the physiological effects of both a single and multiple SUS treatments on short- and long-term neuronal excitability, dendritic morphology and dendritic spine densities in the CA1 region of the hippocampus of wild-type mice (see Fig 1 for experimental design). This allowed us to determine the effect of different SUS treatments in a non-disease state system before eventually moving to a more complicated disease model, where alterations in neuronal function are already present at an early age. For example, reductions in dendritic spine density, AP firing, synaptic activity and long-term potentiation (LTP) have all been reported to occur in amyloid-depositing mouse models of AD [11–15]. In our study using wild-type mice, we found that the different SUS treatment regimes had no deleterious effect on neuronal function or morphology. In addition to this we made the interesting observation that repeated SUS treatments prevented reductions in the dendritic complexity and length of CA1 pyramidal neurons that occur in age-matched sham-treated wild-type mice over the course of three months, while a reduction in dendritic spine density was not halted. Taken together, these findings suggest that multiple SUS treatments ameliorate a reduction in the total number of dendritic spines per neuron. A more extensive follow-up study will determine, whether SUS treatments improves cognition in aging mice and what the underlying mechanism is of such an effect.

## Materials and Methods

### Ethics statement

All experimental procedures in this study were conducted under the guidelines of the Australian Code of Practice for the Care and Use of Animals for Scientific Purposes and were approved by the University of Queensland Animal Ethics Committee (QBI/412/14/NHMRC; QBI/027/12/NHMRC). Mice were maintained on a 12-hour light/dark cycle and housed in a PC2 facility with *ad libitum* access to food and water. Female mice were used to in this study to minimize unused animals in our breeding colony in accordance with the guidelines of the Australian Code of Practice for the Care and Use of Animals for Scientific Purposes. To rule out a confounding effect of the estrous cycle, the group-housed female mice that were 4 months old when our study was initiated were separated from male mice and were not in a breeding rotation for at least 2 months prior to and during experimentation. According to the Lee-Boot effect this would push the female mice into an anestrous state,; however, vaginal lavages were not performed to quantify this.

### Animal preparation for scanning ultrasound (SUS) treatment

A total of 24 female C57Bl/6 mice (3 mice per sham and SUS group for each of the 4 experimental protocols), aged 4 months at the beginning of our proof-of-concept study, were anaesthetized by intraperitoneal injection with ketamine (100 mg/kg, Provet) and xylazine (10 mg/kg, Ilium) and the head hair was removed by shaving and depilatory cream. Mice were injected retro-orbitally with an in-house prepared microbubble solution at 1 μl/g body weight, placed in an immobilizing head frame (Narishige), and an ultrasound transducer was coupled to the head using ultrasound gel [1] (Fig 1A). Following random allocation to a treatment group, animals were treated with a single SUS treatment and analyzed 2 and 24 hours later (Fig 1B), respectively, or received six SUS treatments once weekly for six weeks, and were analyzed after the sixth treatment or were aged for 3 months before final analysis (Fig 1C). Sham-treated mice were used as a control, undergoing all injections and placement under the ultrasound transducer, although no ultrasound was emitted. Mice that received multiple SUS/sham treatments were, after aging for one week or 3 months following the final treatment, 6 and 9 months old, respectively, when the electrophysiological recordings and dendritic analysis were performed (Fig 1D–1F).

### Generation of microbubbles

In-house prepared microbubbles comprising a phospholipid shell and octa-fluoropropane gas core were used [1]. DSPC and DSPE-PEG2000 (Avanti Polar Lipids) at a 9:1 molar ratio were dissolved in chloroform (Sigma) and the chloroform solvent was evaporated under vacuum. The dried phospholipid cake was then dissolved in PBS with 10% glycerol to a concentration of 1 mg lipid/ml and heated to 55°C in a sonicating water bath. The solution was placed in 1.5 ml glass HPLC vials and the air in the vial was replaced with octafluoropropane (Arcadophta). Microbubbles were generated on the day of the experiment by agitating in a dental amalgamator at 4000 rpm for 40 seconds. Microbubbles were polydispersed and were under 10 μm in size at a concentration of 1-5x10^8^ microbubbles/ml.

### Sonication protocol

Ultrasound was generated by the Therapy Imaging Probe System (TIPS, Philips Research) that is composed of an annular array transducer with a focal length of 80 mm, an 80 mm radius of curvature, an 80 mm spherical shell with a 31 mm central opening, and a motorized 3D positioning system to target and move the transducer in scanning mode. The ultrasound settings used for treatments were 1 MHz centre frequency, 0.7 MPa peak rarefactional pressure applied outside the skull, 10 Hz pulse repetition frequency, 10% duty cycle and 10 ms pulse length. Ultrasound was applied sequentially in a scanning mode by applying it for 6 seconds duration per spot, moving the focus 1.5 mm and repeating the application until the entire brain was treated as described previously [1]. The focus of the transducer had a volume of 1.5 mm x 1.5 mm x 12 mm.

### Brain slice preparation

Brain slices were prepared similar to those described previously [16]. SUS- and sham-treated mice were anaesthetized with 2% isoflurane (Attane) and transcardially perfused with cold cutting solution comprising (in mM) 125 choline-Cl, 2.5 KCl, 0.4 CaCl_2_, 6 MgCl_2_, 1.25 NaH_2_PO_4_, 26 NaHCO_3_ and 20 D-glucose saturated with carbogen gas (95% O_2_/5% CO_2_). Mice were then decapitated and the brain quickly removed. 300 μm coronal hippocampal brain slices, from between approximately -1.34 to -2.54 mm of the Bregma, were cut on a vibratome (VT1000S, Leica). Slices were rested for 30 minutes at 35°C and then at room temperature (RT) for at least 30 minutes prior to recordings, which were conducted by an experimenter who was blinded to treatment [16, 17].

### Whole-cell patch-clamp electrophysiology

Slices were transferred to a submerged recording chamber on an upright microscope (SliceScope Pro 1000; Scientifica) and perfused with an oxygenated recording artificial cerebrospinal fluid solution comprising (in mM) 125 NaCl, 2.5 KCl, 2 CaCl_2_, 2 MgCl_2_, 1.25 NaH_2_PO_4_, 26 NaHCO_3_, 10 D-glucose at 32°C. CA1 pyramidal neurons were identified visually using infrared-oblique illumination microscopy using a 40x water-immersion objective (Olympus) and a CCD camera (Jenoptik, Optical Systems GmbH). Whole-cell patch-clamp recordings were made using a micro-manipulator (Scientifica) and an Axon MultiClamp 700B patch-clamp amplifier (Molecular Devices). Data were acquired using pClamp software (v10; MDS) with a sampling rate of 50 kHz after Bessel filtering at 10 kHz (Digidata 1440a; Axon). Patch pipettes (4–7 MΩ; GC150F-10; Harvard Instruments) were pulled using a micropipette puller (PC-10; Narishige) and were filled with an internal solution containing (in mM) 125 K-gluconate, 5 KCl, 2 MgCl_2_.6H_2_0, 10 HEPES, 4 ATP-Mg, 0.3 GTP-Na, 10 phosphocreatine, 10 EGTA and 0.2% biocytin (pH 7.24 and 291 mOsm). The neuronal capacitance, input resistance and time constant were determined in current-clamp mode with the cells held at -70 mV. Input resistance (*R*_m_) was calculated as the slope of the linear fit of the voltage-current plot between a -20 and +20 pA, 400 ms current injection. Time constant (τ) was calculated from the voltage decay (1-1/e) that occurred from a -60 pA, 400 ms current injection. Capacitance (C_m_) was then calculated according to the formulae: τ = *R*_m_C_m_. Neuronal excitability was determined in current-clamp mode, with a holding current injected to maintain the membrane potential at approximately -70 mV; current steps were then injected (-60 to 320 pA pulses in 20 pA amplitude steps, 400 ms step duration) to generate action potential (AP) firing [17].

### Data analysis

Data were analysed blinded to treatment group using Axograph X software (Axograph). Raw traces were normalized by baseline subtraction and input-output relationships were created by determining the number of APs that were generated for each injected current step (-60 to 320 pA in steps of 20pA). Individual APs were identified using a 50 mV/ms threshold. The integrated AP firing was used to compare the AP firing of neurons and was determined by calculating the areas under the curve from individual input-output relationships. The AP amplitude and afterhyperpolarization (AHP) were calculated relative to threshold. The AP rise-time was calculated as the time between 10–90% maximal AP amplitude. The AP half-width was determined at 50% of maximal AP amplitude. The sag potential was calculated by subtracting the membrane voltage at the beginning of a -60 pA, 400 ms hyperpolarizing current step from the steady state. AP adaptation was determined by comparing the amplitude and frequency of the first and tenth AP fired during injection of a 320 pA, 400 ms duration current step.

### Neuron recovery and immunohistochemistry

Following electrophysiological recordings, slices were fixed in cold 4% paraformaldehyde overnight at RT, after which they were washed three times for 10 minutes with 1x PBS solution. They were then incubated for 1 hour in a buffering solution containing 3% BSA, 50 μM glycine, 0.05% sodium azide and 0.3% Triton X-100 in PBS at RT. Following three 10 minute washes with PBS, slices were coupled with Streptavidin to Alexa 594 overnight (1:2,000; Life Technologies) in the presence of 0.1% Triton X-100 and 0.05% sodium azide in PBS at RT. After a final three washes with PBS, slices were cover-slipped with Vectashield mounting medium (H-1400; Vector Laboratories Inc.) and stored at 4°C [17].

### Image acquisition and neuron tracing

The dendritic morphology of recovered neurons was imaged using a laser-scanning confocal microscope (LSM710; Zeiss) with a 20x air objective (0.8 NA; Olympus) and acquired using Zen 2012 software (Zeiss). A spinning-disk confocal system (Marianas; 3I, Inc.) consisting of an Axio Observer Z1 (Zeiss) equipped with a CSU-W1 spinning-disk head (Yokogawa Corporation of America) and an ORCA-Flash4.0 v2 sCMOS camera (Hamamatsu Photonics) with a 100x oil-immersion objective (1.4 NA; Zeiss) was used to image dendritic spines with Nyquist sampling using Slidebook 6 software (3i Inc.) so that each image was acquired with 16 bits per pixel and an individual pixel resolution of 0.063x0.063x0.13 μm^3^ per pixel. Dendritic spine images were then deconvolved using Huygens Professional software (SVI). Recovered neurons were manually traced and dendritic spines were detected using automated analysis within Neurolucida software (MBF Bioscience). If a dendritic tree was not successfully filled with biocytin it was excluded from analysis; two basal trees of neurons were excluded from analysis on this criterion. Spine density analysis was performed on all successfully recovered neurons where the dendritic spines were obviously present upon visual examination. Of these identified neurons, three randomly selected separate dendritic branches per neuron, with an average length of 600 μm that originated from the main apical branch, were imaged and underwent spine density quantification to create an average spine density per neuron using automated detection in Neurolucida software (MBF Bioscience). Neuroexplorer software (MBF Bioscience) was then used to calculate dendritic morphology and dendritic spine densities.

### Statistical analysis

Statistical analysis was performed using GraphPad Prism software (v6; GraphPad Software Inc.). Statistical comparisons between SUS- and sham-treated groups were made using a two-tailed unpaired Mann-Whitney U-test. A two-way ANOVA with a Bonferroni’s post hoc test was used to compare dendritic branch order of SUS- and sham-treated groups. An alpha value of 0.05 was used in all cases. Data are presented as mean ± SEM and as individual data points.

## Results

### A single SUS treatment does not alter the short-term excitability of hippocampal CA1 neurons

**Fig 2:**
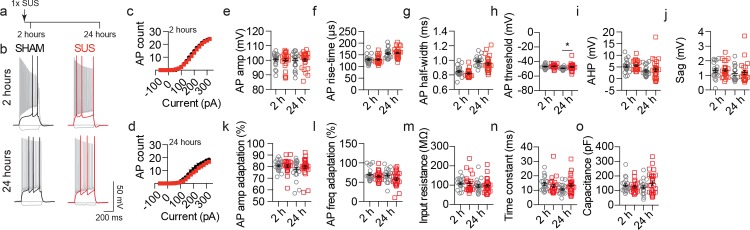
A single SUS treatment does not alter CA1 pyramidal neuronal excitability at short time-points. (a) Overview of the experimental design of the single SUS/sham treatment. Arrows indicate number of treatments. (b) Representative traces of APs generated by injection of current steps (-60, 0, threshold and 320 pA). (c, d) Input-output relationships from neurons in sham- (black circles) and SUS-treated mice (red squares) recorded (c) 2 hours (2 h, p = 0.56, SUS 171.1 ± 13.7 a.u., sham 180.4 ± 13.8 a.u.) or (d) 24 hours (24 h, p = 0.33, SUS 109.9 ± 10.6 a.u., sham 132.6 ± 14.6 a.u.) following a single treatment. Quantification of (e) AP amplitude (2 h, p = 0.93, SUS 99.98 ± 1.2 mV, sham 100.4 ± 0.98 mV; 24 h, p = 0.89, SUS 100.3 ± 0.99 mV, sham 99.99 ± 1.3 mV), (f) rise-time (2 h, p = 0.89, SUS 129.5 ± 3.5 μs, sham 129.4 ± 4.1 μs; 24 h, p = 0.58, SUS 158.1 ± 3.90 μs, sham 155.8 ± 4.61 μs), (g) half-width (2 h, p = 0.09, SUS 0.82 ± 0.01 ms, sham 0.85 ± 0.02 ms; 24 h, p = 0.06, SUS 0.95 ± 0.02 ms, sham 0.99 ± 0.01 ms), (h) AP threshold (2 h, p = 0.42, SUS -46.4 ± 0.53 mV, sham -47.0 ± 0.63 mV; 24 h, p = 0.0147, SUS -47.5 ± 0.82, sham -49.2 ± 0.4 mV) (**i**) AHP (2 h, p = 0.82, SUS 5.6 ± 0.42 mV, sham 5.3 ± 0.58 mV; 24 h, p = 0.80, SUS 4.1 ± 0.86 mV, sham 3.6 ± 0.57 mV), (i) sag (2 h, p = 0.93, SUS 1.36 ± 0.11 mV, sham 1.33 ± 0.12 mV; 24 h, p = 0.75, SUS 1.2 ± 0.14 mV, sham 1.1 ± 0.13 mV), (k) AP amplitude adaptation (2 h, p = 0.84, SUS 80.57 ± 1.23%, sham 80.7 ± 0.90%; 24 h, p = 0.39, SUS 89.2 ± 1.48%, sham 77.5 ± 1.95%), (l) AP frequency adaptation (2 h, p = 0.30, SUS 65.1 ± 2.67%, sham 70.7 ± 3.49%; 24 h, p = 0.10, SUS 58.1 ± 3.83%, sham 67.4 ± 3.81%), (m) input resistance (2 h, p = 0.39, SUS 109.2 ± 9.3 MΩ, sham 109.8 ± 6.2 MΩ; 24 h, p = 0.89, SUS 96.87 ± 6.5 MΩ, sham 95.5 ± 7.3 MΩ), (n) time constant (2 h, p = 0.14, SUS 13.02 ± 1.2 ms, sham 14.5 ± 1.0 ms; 24 h, p = 0.11, SUS 13.45 ± 1.1 ms, sham 10.81 ± 0.75 ms) and (o) capacitance (2 h, p = 0.43, SUS 124.4 ± 7.91 pF, sham 136.3 ± 10.42 pF; 24 h, p = 0.22, SUS 151.7 ± 13.94 pF, sham 122.7 ± 10.62 pF). Data are presented as mean ± SEM and as individual data points. *p<0.05. Sample size: 2 hour time-point—SUS n = 23 neurons and sham n = 20 neurons. 24 hour time-point—SUS n = 28 neurons and sham n = 20 neurons.

In our analysis we focused on CA1 pyramidal neurons as this region and cell type has previously been reported to play an important role in hippocampal-dependent memory tasks [18, 19], the function of which was improved following SUS treatment in an amyloid-depositing mouse model of AD [1]. To investigate the short-term effects of SUS on neuronal excitability we first performed whole-cell patch-clamp recordings in brain slices cut from mice 2 or 24 hours after a single SUS or sham treatment (Fig 2A). No change in AP firing was observed at either time-point in neurons from SUS-treated mice compared to sham controls (Fig 2B–2D). In addition, AP kinetics was not affected by SUS treatment, as AP amplitude, rise-time, half-width, AHP, sag potential and AP amplitude and frequency adaptation were not altered compared to sham-treated neurons (Fig 2E–2G and 2I–2L). A subtle, yet statistically significant, depolarized shift in the threshold of AP generation was observed only at 24 hours after SUS treatment, however this was not sufficient to alter AP firing (Fig 2D and 2H). Furthermore, no change in the passive properties of the recorded CA1 neurons was observed at either of the two time-points (Fig 2M and 2N).

### Multiple SUS treatments do not impair the long-term excitability of CA1 pyramidal neurons

**Fig 3:**
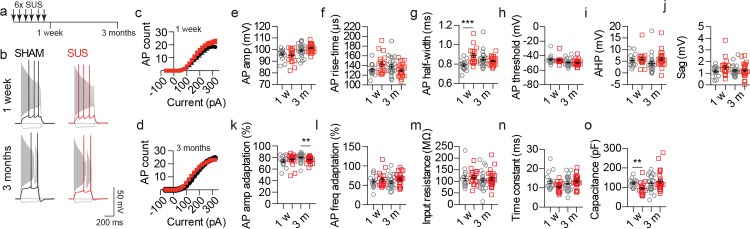
CA1 neuronal excitability is not impaired by multiple SUS treatments even after long time periods. (a) Schematic of the experimental design of multiple treatments. (b) Representative traces of APs generated by injection of current steps (-60, 0, threshold and 320 pA). (c, d) Input-output relationships from sham- and SUS-treated neurons recorded (**c**) 1 week (1 w, p = 0.39, SUS 182.5 ± 18.2 a.u., sham 155.7 ± 22.7 a.u.) or (d) 3 months (3 m, p = 0.62, SUS 204.8 ± 12 a.u., sham 193.4 ± 18.78 a.u.) after the end of treatment. Quantification of (e) AP amplitude (1 w, p = 0.88, SUS 95.1 ± 1.3 mV, sham 95.6 ± 1.3 mV; 3 m, p = 0.67, SUS 101.4 ± 0.68 mV, sham 100.1 ± 1.3 mV), (f) rise-time (1 w, p = 0.13, SUS 142.9 ± 4.5 μs, sham 132.1 ± 3.6 μs; 3 m, p = 0.23, SUS 130.2 ± 2.52 μs, sham 137.2 ± 4.43 μs), (g) half-width (1 w, p<0.0001, SUS 0.89 ± 0.02 ms, sham 0.79 ± 0.02 ms; 3 m, p = 0.96, SUS 0.83 ± 0.008 ms, sham 0.84 ± 0.02 ms), (h) AP threshold (1 w, p = 0.63, SUS -46.2 ± 0.99 mV, sham -45.4 ± 1.58 mV; 3 m, p = 0.93, SUS -49.7 ± 0.75 mV, sham -49.4 ± 0.95 mV), (i) AHP (1 w, p = 0.34, SUS 5.8 ± 0.69 mV, sham 5.1 ± 0.69 mV; 3 m, p = 0.0011, SUS 5.9 ± 0.62 mV, sham 3.8 ± 0.94 mV), (j) sag (1 w, p = 0.10, SUS 1.5 ± 0.16 mV, sham 1.2 ± 0.13 mV; 3 m, p>0.99, SUS 1.3 ± 0.11 mV, sham 1.3 ± 0.11 mV), (k) AP amplitude adaptation (1 w, p = 0.21, SUS 76.3 ± 2.32%, sham 73.8 ± 2.33%; 3 m, p = 0.0081, SUS 76.6 ± 0.94%, sham 80.0 ± 1.47%), (l) AP frequency adaptation (1 w, p = 0.71, SUS 64.1 ± 4.23%, sham 60.0 ± 4.37%; 3 m, p = 0.08, SUS 66.3 ± 2.90%, sham 58.5 ± 3.52%), (m) input resistance (1 w, p = 0.28, SUS 117.5 ± 6.97 MΩ, sham 112.5 ± 10.69 MΩ; 3 m, p = 0.58, SUS 110.7 ± 6.53 MΩ, sham 105.8 ± 8.07 MΩ), (n) time constant (1 w, p = 0.08, SUS 10.5 ± 0.55 ms, sham 13.5 ± 1.16 ms; 3 m, p = 0.31, SUS 12.3 ± 0.62 ms, sham 12.4 ± 0.77 ms) and (o) capacitance (1 w, p = 0.0016, SUS 94.0 ± 6.88 pF, sham 122.2 ± 5.52 pF; 3 m, p = 0.49, SUS 131.3 ± 9.40 pF, sham 118.9 ± 2.82 pF). Data are presented as mean ± SEM and as individual data points. **p<0.01 and ***p<0.001. Sample size: one week time-point—SUS n = 19 neurons and sham n = 15 neurons. 3 month time-point—SUS n = 27 neurons and sham n = 24 neurons.

Although neuronal excitability was not impaired following a single SUS treatment, it was also important to determine if multiple SUS treatments caused a long-term impairment. Wild-type mice were therefore treated with six weekly SUS/sham treatments and allowed to age for either one week or 3 months before patch-clamp recordings were done from CA1 pyramidal neurons (Fig 3A). Similar to the single treatment groups, AP firing was not altered at either one week or 3 months compared to sham controls (Fig 3B–3D). Multiple SUS treatments resulted in an increase in the AP half-width only at the one week time-point and a small reduction in the AP amplitude adaptation only at the 3 month time-point was observed (Fig 3G and 3K), neither of which changed AP firing (Fig 3C and 3D). No other parameter of AP kinetics (Fig 3E, 3F, 3H–3J and 3L) or the passive properties (Fig 3M and 3N) of the recorded neurons was altered by SUS treatment at either time-point compared to sham controls.

### Multiple SUS treatments prevent CA1 dendrite loss

**Fig 4:**
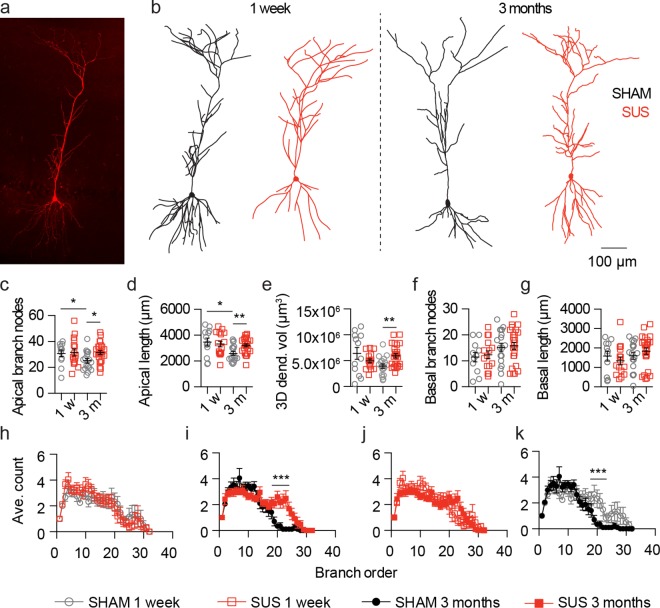
Multiple SUS treatments prevent dendrite loss of CA1 pyramidal neurons. (a) Fluorescent z-projection of a recovered fluorescently labeled neuron used for morphometric analysis. (b) Representative 2D projection of 3D reconstructions of recorded neurons from SUS- and sham-treated mice. (c-g) Quantification of (c) the number of apical dendrite branch nodes (SUS versus sham 1 w, p = 0.88, SUS 31.6 ± 2.71 nodes, sham 30.8 ± 2.43 nodes; SUS versus sham 3 m, p = 0.0119, SUS 31.4 ± 1.52 nodes, sham 25.1 ± 1.73 nodes; 1 w versus 3 m sham, p = 0.028, 1 w 30.8 ± 2.43 nodes, 3 m 25.1 ± 1.73 nodes; 1 w versus 3 m SUS, p = 0.69, 1 w 31.6 ± 2.71 nodes, 3 m 31.4 ± 1.52 nodes), (d) total apical dendrite length (SUS versus sham 1 w, p = 0.66, SUS 3312 ± 223.7 μm, sham 3465 ± 308.2 μm; SUS versus sham 3 m, p = 0.0017, SUS 3210 ± 118.1 μm, sham 2592 ± 143.6 μm; 1 w versus 3 mo sham, p = 0.0346, 1 w 3465 ± 308.2 μm, 3 m 2592 ± 143.6 μm; 1 w versus 3 m SUS, p = 0.76, 1 w 3312 ± 223.7 μm, 3 m 3210 ± 118.1 μm), (e) 3D dendritic tree volume (SUS versus sham 1 w, p = 0.35, SUS 5.00x10^6^ ± 0.39x10^6^ μm^3^, sham 6.4x10^6^ ± 1.15x10^6^ μm^3^; SUS versus sham 3 m, p = 0.0038, SUS 6.0x10^6^ ± 0.42x10^6^ μm^3^, sham 4.0x10^6^ ± 0.42x10^6^ μm^3^; 1 w versus 3 m sham, p = 0.11, 1 w sham 6.4x10^6^ ± 1.15x10^6^ μm^3^, 3 m 4.0x10^6^ ± 0.42x10^6^ μm^3^; 1 w versus 3 m SUS, p = 0.17, 1 w 5.00x10^6^ ± 0.39x10^6^ μm^3^, 3 m 6.0x10^6^ ± 0.42x10^6^ μm^3^) (f) the number of basal dendrite branch nodes (1 w, p = 0.77, SUS 12.3 ± 1.53 nodes, sham 11.5 ± 1.84 nodes; 3 m, p = 0.81, 15.8 ± 1.37 nodes, sham 15.2 ± 1.65 nodes), (g) total basal dendrite length (1 w, p = 0.42, SUS 1335 ± 202.8 μm, sham 1584 ± 256.9 μm; 3 m, p = 0.33, SUS 1834 ± 167.5 μm, sham 1596 ± 176.6 μm). (h-k) Quantification of the dendritic branch order (h, SUS versus sham 1 w, p = 0.063, *F*_(1, 832)_ = 0.23; i, SUS versus sham 3 m, p = 0.0001, *F*_(1, 1280)_ = 17.93; j, 1 w versus 3 m SUS, p = 0.41, *F*_(1, 1248)_ = 0.68; k, 1 w versus 3 m sham, p = 0.0001, *F*_(1, 864)_ = 15.26). Data are presented as mean ± SEM and as individual data points. *p<0.05, **p<0.01 and ***p<0.001. Sample size: one week time-point—SUS n = 16 neurons and sham n = 12 neurons. 3 month time-point—SUS n = 25 neurons and sham n = 18 neurons.

Having demonstrated that SUS treatment had little effect on neuronal AP firing, we next investigated whether it affected neuronal structure. Using immunohistochemistry following patch-clamp recordings, we fluorescently labelled neurons filled with biocytin (Fig 4A) and manually reconstructed their full dendritic morphology in 3D (Fig 4B). This allowed us to successfully recover 83% of all recorded neurons for a morphological analysis. At the one week time-point, no change was observed in the number of branch nodes, the total dendritic length, the 3D dendritic volume and the dendritic branch order of the apical dendritic tree of SUS- compared to sham-treated CA1 neurons, a neuronal cell-type that predominately receives synaptic input from the CA3 region along the Schaffer collateral pathway (Fig 4C–4E and 4H). Interestingly, at the 3 month time-point, in sham-treated compared with SUS-treated neurons, the number of branch nodes, the total dendritic tree length, the 3D dendritic volume, and the dendritic branch order of the apical dendritic trees were all reduced (Fig 4C–4E and 4I). When the structure of the apical dendritic tree at 3 months after SUS treatments was compared with that observed at the one week time-point it was not altered (Fig 4C–4E and 4J). When sham-treated neurons at the 3 month time-point were compared to the one week time-point, the number of branch nodes, the total dendritic tree length and the dendritic branch order were also all reduced (Fig 4C, 4D and 4K). The structure of the basal dendritic tree of SUS- and sham-treated CA1 neurons was not altered at either time-point (Fig 4F and 4G). Together, these data suggest that the observed differences between the SUS- and sham-treated neurons at the 3 month post treatment time-point result from a prevention of an age-associated reduction in the apical dendritic tree.

### CA1 dendritic spine density is not altered by SUS treatment

**Fig 5:**
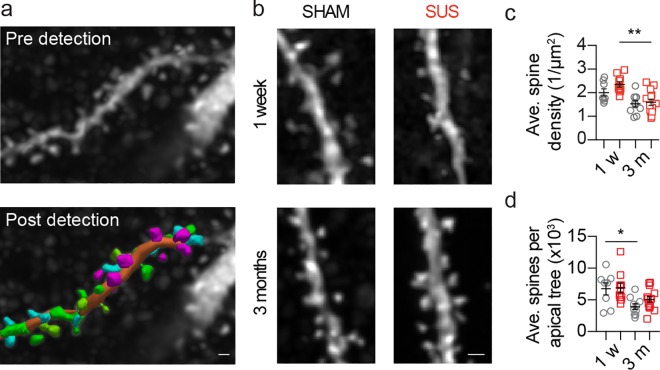
The dendritic density of CA1 neurons is not altered following multiple SUS treatments. (a) A representative fluorescent image of an apical dendritic branch and spines (top) demonstrating the 3D dendritic spine identification (bottom). (b) Representative confocal z-projections of spines from SUS and sham neurons. Graphs depict (c) the average apical dendritic spine densities (SUS versus sham 1 w, p = 0.10, SUS 2.4 ± 0.11 1/μm^2^, sham 2.0 ± 0.15 μm^2^; SUS versus sham 3 m, p = 0.84, SUS 1.6 ± 0.13 μm^2^, sham 1.5 ± 0.13 μm^2^; 1 w versus 3 m sham, p = 0.10, 1 w 2.0 ± 0.15 μm^2^, 3 m 1.5 ± 0.13 μm^2^; 1 w versus 3 m SUS, p = 0.0012, 1 w 2.4 ± 0.11 1/μm^2^, 3 m 1.6 ± 0.13 μm^2^) and (d) the average number of apical dendritic spines (SUS versus sham 1 w, p = 0.82, SUS 6871 ± 765.3 spines, sham 6749 ± 948.8 spines; SUS versus sham 3 m, p = 0.11, SUS 5077 ± 450.1 spines, sham 3937 ± 439.8 spines; 1 w versus 3 m sham, p = 0.0343, 1 w 6749 ± 948.8 spines, 3 m 3937 ± 439.8 spines; 1 w versus 3 m SUS, p = 0.07, 1 w 6871 ± 765.3 spines, 3 m 5077 ± 450.1 spines). Data are presented as mean ± SEM and as individual data points. *p<0.05 and ***p<0.001. Sample size: one week time-point—SUS n = 8 neurons and sham n = 10 neurons. 3 month time-point—SUS n = 14 neurons and sham n = 10 neurons. Scale bar: 0.5 μm.

After demonstrating that multiple SUS treatments prevent reductions in apical dendritic tree length and complexity, without altering the basal dendritic tree, we investigated if the density of apical dendritic spines was affected by SUS treatment. A total of 126 apical dendrites from 42 neurons (63% of neurons that underwent morphometric analysis) were imaged using high-resolution spinning disk confocal microscopy, resulting in a total of 11,912 individual dendritic spines being counted in 3D using automated detection (Fig 5A). No change in the density of apical dendritic spines was observed when SUS- and sham-treated neurons were assessed one week after treatment, and also when the two groups were compared 3 months after treatment (Fig 5B and 5C), However, the spine density in the apical tree of SUS treated neurons was reduced at the 3 month compared to the one week time-point. Although, considering that the average number of spines per apical tree was only reduced at one week time-point compared to 3 months after sham treatments (Fig 5D), this suggests that SUS treatment ameliorates the loss of the total dendritic spines per CA1 pyramidal neuron in our experimental paradigm.

## Discussion

In the present study that had initially been designed as a safety study, we found no altered firing of CA1 pyramidal neurons after either a single SUS treatment or six repeated weekly treatments of wild-type mice. This supports a body of literature that indicates that because ultrasound is tunable, combining sonication with microbubbles to transiently open the BBB can be achieved without overt damage to the brain. Interestingly, we found that repeated SUS treatments prevented the reduction in length and complexity of the apical dendritic tree that occurred in wild-type mice over a three month time period, without altering dendritic spine density. Collectively, these data not only provide further supporting evidence that SUS treatment does not harm the brain but that it may also have potential beneficial affects by preserving the dendritic tree structure during the ageing process.

Age-associated reductions in the structure of neuronal dendritic trees have previously been reported in a range of brain areas and species; including the prefrontal, superior temporal and precentral cortices in humans and non-human primates, dogs and mice [20–26]. However, our understanding of changes in dendritic tree arborization in the hippocampus is less advanced, where both increases and decreases in CA1 pyramidal dendritic length and complexity have previously been reported [27, 28]. While a number of differences exist between these reports and the current work, perhaps the most important is the duration of ageing over which changes in dendritic tree structure was quantified (approximately 1.5 years versus 3 months in the current study). This is a limitation of the current study when evaluating changes in dendritic structure associated with ageing. Further experimentation will be required to assess changes in dendritic tree structure over longer time periods. Despite this, it is evident that multiple SUS treatments are able to prevent reductions in dendritic structure. Considering (i) that reductions in CA1 pyramidal neuron dendritic tree structure have been reported to be sufficient to cause abnormal AP firing [14], (ii) that dendritic branches, rather than individual synapses, are the primary site for protein synthesis-dependent LTP [29], (iii) that reductions in LTP induction and postsynaptic potentials recorded *in vitro* and *in vivo* have been reported following stimulation of CA1 pyramidal neuron inputs in old animals [30, 31], and (iv) that these reductions in synaptic activity are considered causative in impairing performance in hippocampal-dependent memory tasks [32, 33], the preservation of the CA1 pyramidal neuron apical dendritic tree structure following multiple SUS treatments may reflect an amelioration of the reduced synaptic activity that occurs during the ageing process. However, this will require further experimentation to validate. Furthermore, while we have not observed a change in neuronal excitability from patch-clamp recordings performed at the soma, we cannot preclude that the changes in dendritic tree structure we describe may affect physiological functions that occur within the dendritic tree, such as the integration of synaptic inputs, calcium and N-methyl-D-aspartate (NMDA) spikes [34].

Also, the question of how SUS preserves dendritic structure remains to be determined. One possibility is that microglia may play a role, because SUS treatment has previously been reported to activate this cell-type in APP23 and TgCRND8 amyloid-depositing mouse models as well as in wild-type mice [1, 5]. Microglia constantly probe their local environment and secrete factors that alter neuronal signalling. During activation they can also modify synaptic connections, the key mediators of learning and memory [35], by increasing the expression of neurotrophins such as brain-derived neurotrophic factor (BDNF) [36]. In fact, a recent study has reported that microglia mediate synapse loss at an early time-point in AD mouse models [37]. One possible mechanism for the neurotrophic effect of SUS may therefore be the delivery of endogenously circulating neurotrophic factors from the blood to the brain. In addition, ultrasound-mediated opening of the BBB increases the number of NeuN/BrdU-positive neurons in the dentate gyrus of the hippocampus of 4 month-old wild-type mice [38], supporting the concept that SUS treatment is neurotrophic and maintains synaptic connections. Similarly, in TgCRND8 mice, ultrasound treatment has been reported to increase the number of newborn doublecortin-positive cells, as well as the length and complexity of their dendritic trees within the dentate gyrus [39]. Furthermore, ultrasound waves by themselves, without the need for BBB opening, may also contribute to the observed preservation of dendritic structure, as increased BDNF expression has been reported following ultrasound application [40]. Other neurotrophic factors such as glial cell -derived neurotrophic factor (GDNF) and vascular endothelial growth factor (VEGF) have also been linked to improve memory performance in rats following ultrasound treatment [41]. While we have shown a preservation of dendritic structure in wild-type mice, further work is required to determine the contribution of microglia and blood-borne elements to this effect.

In conclusion, we demonstrate here that SUS treatment can be safely applied to the rodent brain as determined by the absence of changes in neuronal AP firing. Furthermore, in addition to the previously reported ability of SUS to clear amyloid from the brains of APP23 mice, multiple treatments prevent a loss of hippocampal dendritic length and complexity in wild-type mice that occurs over a three month period, suggesting that this treatment may ameliorate reductions in synaptic activity and cognitive decline that can occur with age.
